# Diving Into Reef Ecosystems for Land-Agriculture Solutions: Coral Microbiota Can Alleviate Salt Stress During Germination and Photosynthesis in Terrestrial Plants

**DOI:** 10.3389/fpls.2020.00648

**Published:** 2020-05-25

**Authors:** Héctor Ocampo-Alvarez, Iván D. Meza-Canales, Carolina Mateos-Salmón, Eduardo Rios-Jara, Fabián A. Rodríguez-Zaragoza, Celia Robles-Murguía, Alejandro Muñoz-Urias, Rosalba Mireya Hernández-Herrera, Francisco Javier Choix-Ley, Amayaly Becerril-Espinosa

**Affiliations:** ^1^Laboratorio de Ecología Molecular, Microbiología y Taxonomía, Departamento de Ecología, Centro Universitario de Ciencias Biológicas y Agropecuarias, Universidad de Guadalajara, Zapopan, Mexico; ^2^Laboratorio de Evolución de Sistemas Ecológicos, Departamento de Ecología, Centro Universitario de Ciencias Biológicas y Agropecuarias, Universidad de Guadalajara, Zapopan, Mexico; ^3^Laboratorio de Biología Molecular, Genómica y Proteómica, Instituto Transdisciplinar de Investigación y Servicios, Universidad de Guadalajara, Zapopan, Mexico; ^4^Laboratorio de Investigación en Biotecnología, Departamento de Botánica y Zoología, Centro Universitario de Ciencias Biológicas y Agropecuarias, Universidad de Guadalajara, Zapopan, Mexico; ^5^CONACYT, Departamento de Ingeniería Química, CUCEI, Universidad de Guadalajara, Guadalajara, Mexico; ^6^CONACYT, Departamento de Ecología, Centro Universitario de Ciencias Biológicas y Agropecuarias, Universidad de Guadalajara, Zapopan, Mexico

**Keywords:** coral-bacteria interactions, actinobacteria, plant biostimulants, *Salinispora* symbiont, agricultural solutions, plant growth, saline stress

## Abstract

From their chemical nature to their ecological interactions, coral reef ecosystems have a lot in common with highly productive terrestrial ecosystems. While plants are responsible for primary production in the terrestrial sphere, the photosynthetic endosymbionts of corals are the key producers in reef communities. As in plants, coral microbiota have been suggested to stimulate the growth and physiological performance of the photosynthetic endosymbionts that provide energy sources to the coral. Among them, actinobacteria are some of the most probable candidates. To explore the potential of coral actinobacteria as plant biostimulants, we have analyzed the activity of *Salinispora* strains isolated from the corals *Porites lobata* and *Porites panamensis*, which were identified as *Salinispora arenicola* by 16S rRNA sequencing. We evaluated the effects of this microorganism on the germination, plant growth, and photosynthetic response of wild tobacco (*Nicotiana attenuata*) under a saline regime. We identified protective activity of this actinobacteria on seed germination and photosynthetic performance under natural light conditions. Further insights into the possible mechanism showed an endophytic-like symbiosis between *N. attenuata* roots and *S. arenicola* and 1-aminocyclopropane-1-carboxylate (ACC) deaminase activity by *S. arenicola.* We discuss these findings in the context of relevant ecological and physiological responses and biotechnological potential. Overall, our results will contribute to the development of novel biotechnologies to cope with plant growth under saline stress. Our study highlights the importance of understanding marine ecological interactions for the development of novel, strategic, and sustainable agricultural solutions.

## Introduction

With the expectation that future changes in demographic and climatic scenarios will put additional pressure on the environment and agricultural production worldwide, the need to develop sustainable high-quality and high-yield crop alternatives is more important now than ever before (Yamaguchi and Blumwald, 2005). Projections of future environmental changes have created agricultural challenges characterized by increased biotic and abiotic stress (Meena et al., 2017). In particular, soil salinity has become especially important in recent years and is considered to be one of the most pressing abiotic factors threatening agricultural production (Isayenkov and Maathuis, 2019). One promising initiative for a new model of environmentally friendly agriculture is based on converting the natural processes that occur in soil-plant systems into biotechnologies that enhance crop production (Sahu et al., 2018). In this context, a great deal of research has focused on understanding the relationships among plants and soil-borne symbiotic microorganisms that increase the ecological success of plants in their natural habitats (Compant et al., 2019; Kumar and Meena, 2019). Given the ecological advantages offered by these microorganisms and their bioactive compounds, they have been employed as biostimulants to improve growth and yield for multiple plant species (Yakhin et al., 2017).

The most common living biostimulants are microorganisms that either colonize the rhizosphere or live within plant tissues (i.e., endophytic microorganisms) and form close mutualistic relationships. Plants benefit from these microorganisms through multiple mechanisms, such as the provision of growth phytohormones, increased mineral solubilization through pH regulation, molecular nitrogen fixation, and the induction of defense and resistance responses to both biotic and abiotic pressures (Smith et al., 2015; Lata et al., 2018). For instance, saline environmental resistance has been shown to occur due to the activity of the plant-associated bacterial enzyme 1-aminocyclopropane-1-carboxylate (ACC) deaminase. This deaminase prevents ethylene biosynthesis and thus prevents stress-related ethylene-induced responses from affecting plant growth (Singh et al., 2015). Reciprocally, plants share photosynthetically fixed carbon to maintain a community of beneficial microorganisms. As plants are a secure source of photosynthates, any non-photosynthetic organism capable of accessing plant energy in a stable mutualistic interaction is thus conferred an advantage by its associated plant (Smith et al., 2015).

In a sense, reef ecosystems have a lot in common with highly productive terrestrial ecosystems. Reef corals present a high density and diversity of associated and beneficial microorganisms. The best-known microorganism that forms a very close relationship with corals is the symbiotic photosynthetic dinoflagellate *Symbiodinium* spp., which produces most of the food for the coral through photosynthesis (Bourne et al., 2016). However, to fulfill the high demand of photosynthates required by corals in a highly erratic environment, we hypothesize that other related mutualists, such as bacteria, may serve as biostimulants that enhance the capabilities of *Symbiodinium*. There is evidence that aquatic phototrophs, such as microalgae, can establish mutualistic interactions with terrestrial bacteria. Microalgae-bacteria interactions have been artificially induced with soil-borne bacteria strains that possess plant-growth-promoting (PGP) activity in terrestrial environments. For example, the interaction of the soil-borne bacteria *Azospirillum brasilense* with the microalgae *Chlorella sorokiniana* was found to increase photosynthetic activity, population density, and accumulation of cellular compounds in the microalgae (Palacios et al., 2016; Amavizca et al., 2017). This biostimulant activity of *A. brasilense* was further shown to be due to a constant provision of the phytohormone indoleacetic acid (IAA) by the bacteria (De-Bashan et al., 2008; Choix et al., 2014; Palacios et al., 2016).

In a recent study, Ainsworth et al. (2015) reported that actinobacteria share a niche habitat with *Symbiodinium* spp. in coral-gastro-dermal cells. It is also known that actinobacteria found in corals, such as members of the order Frankiales, are capable of fixing nitrogen (Sellstedt and Richau, 2013). Moreover, genomic analysis of marine Actinobacteria genera, such as *Streptomyces* and *Salinispora*, have shown the presence of genes related to the metabolic routes for phytohormone and siderophore production (Penn et al., 2009; Qin et al., 2017), which are properties of interest for terrestrially cultured plants. Actinobacteria is an important microorganism phylum whose members have several biotechnological applications. These bacteria are well recognized for possessing an arsenal of biosynthetic pathways for different secondary metabolites (Kasanah and Triyanto, 2019), including known and putative plant biostimulants (Palaniyandi et al., 2013; Olanrewaju and Babalola, 2019).

Although studies of actinobacteria and biostimulant activity have been carried out mainly with strains isolated from terrestrial sources, such as the rhizosphere of crop plants (El-Tarabily et al., 2009; Anwar et al., 2016; Chen et al., 2018; AbdElgawad et al., 2019), the search for novel actinobacteria functions has recently expanded to other less-explored habitats. Studies have been carried out with organisms isolated from seawater, sediments (i.e., *Saccharopolyspora* and *Streptomyces* strains; Rashad et al., 2015; Nafis et al., 2019), and the rhizospheres of both marsh and mangrove plants (Suksaard et al., 2017; Gong et al., 2018). Like their terrestrial counterparts, some of the marine strains have been found to possess the ability to produce phytohormones, fix nitrogen, solubilize phosphate, produce siderophores, and decrease ethylene overproduction via the enzyme ACC deaminase (Rashad et al., 2015). Furthermore, other marine actinobacteria, such as *Streptomyces*, *Isoptericola*, and *Arthrobacter*, have been found to enhance the germination of *Limonium sinensis* plants in soil with different salinities (Qin et al., 2014). However, to the best of our knowledge, the activity of coral-associated actinobacteria has not yet been explored.

**FIGURE 1:**
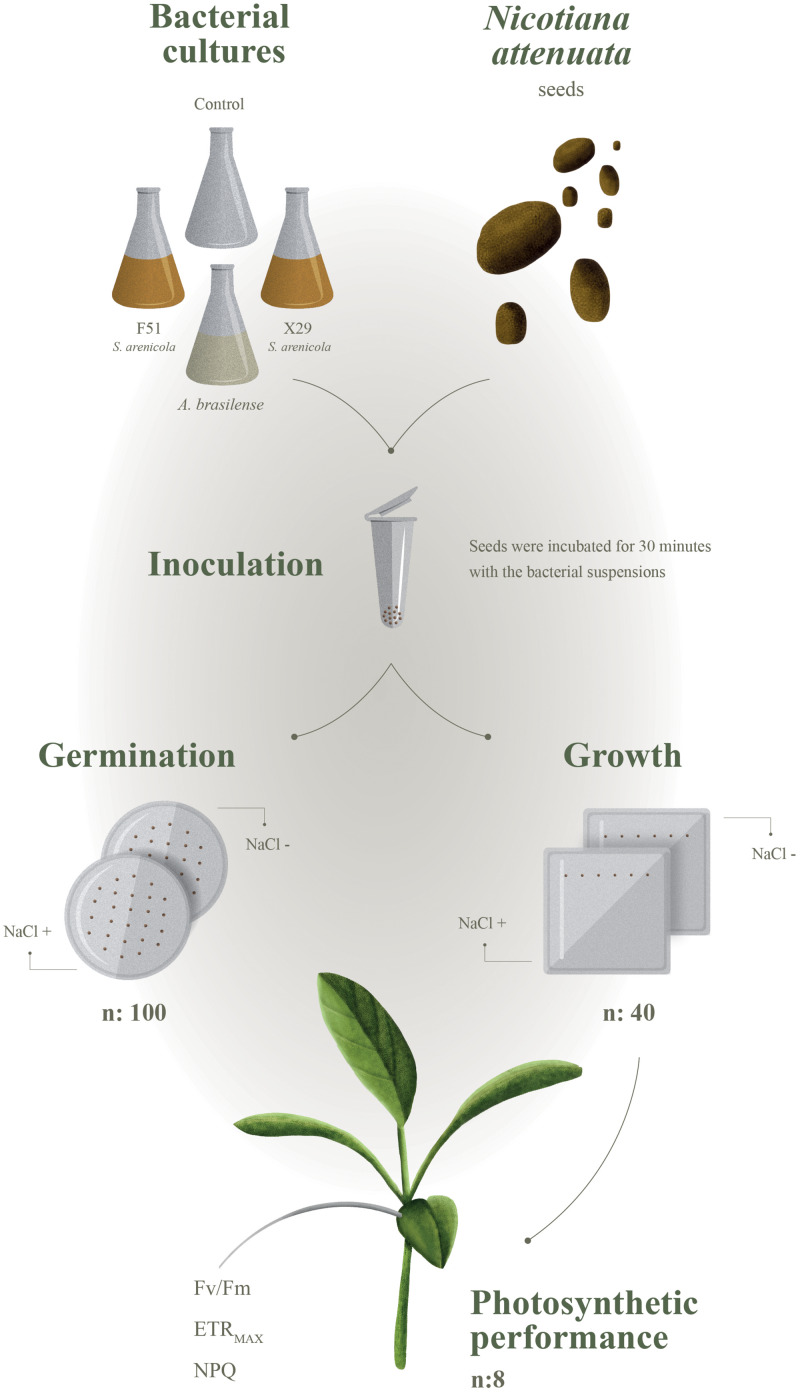
Experimental design to evaluate if *Salinispora* strains can benefit terrestrial plants (*Nicotiana attenuata*) under saline stress.

In this study, we hypothesized that marine actinobacteria associated with coral reefs would support activities that are beneficial for terrestrial plants by enhancing their ability to tolerate abiotic stress, such as increased soil salinity. We tested this hypothesis by isolating actinobacteria from two coral species (*Porites lobata* and *Porites panamensis*) from the tropical central Pacific. We evaluated their ability to act as biostimulants during the germination and early growth of *Nicotiana attenuata* as well as their ability to increase the resistance of this plant to saline stress (Figure 1). *N. attenuata* is a member of the Solanaceae family and is closely related to important crop plants, such as the tomato, whose natural history and responses to environmental stress have been extensively studied (Baldwin et al., 1994).

## Materials and Methods

### Biological Samples

*Salinispora* strains were isolated from two coral species, *P. lobata* and *P. panamensis*. Coral samples were collected by scuba diving in two coral reefs from the tropical central Pacific (19° 5′ 55.21′′ N, 104° 23′ 24.47′′ W and 19° 3′ 28.87′′ N, 104° 15′ 40.25′′ W; Supplementary Figure S1). Coral branches (2 cm) were collected in triplicate from healthy individuals of each species. The collected branch surface was washed several times with sterile seawater to eliminate the mucus layer and epibiont microorganisms. The coral tissue was obtained by airbrushing (80 psi) with 10 mL of sterile seawater. The tissue samples were dried in a laminar flow hood for 72 h and stamped in 10% A1 culture medium (Mincer et al., 2002) supplemented with 100 μg mL^–1^ cycloheximide and 5 μg mL^–1^ gentamicin (Sigma-Aldrich, St. Louis, MO, United States). Strain identification was first carried out using the morphological characteristics of the cultures as well as the seawater requirements for growth and was further confirmed by 16S rRNA gene sequencing (Mincer et al., 2002; Gontang et al., 2007). *Salinispora* strains were preserved on plates with A1 medium until further use. Cultures for seed treatment were grown on 50 mL liquid A1 medium at 210 rpm and 28°C for 8 days.

### 16S rRNA Gene Sequencing and Phylogenetic Analysis

Genomic actinobacteria DNA was extracted using a DNeasy^®^ Blood and Tissue kit (Cat. No. 69506; Qiagen Corp., Germany) according to the methods described by Gontang et al. (2007). For the amplification of the 16S rRNA gene, the primers FC27 (5′-AGAGTTTGATCCTGGCTCAG-3′) and RC1492 (5′-TACGGCTACCTTGTTACGACTT-3′) were selected, and a Dream Taq Green Master Mix (2X) kit was used following the protocols of the manufacturer (#K1081, Thermo Scientific, Vilnius, Lithuania). The PCR products were purified with a Wizard^®^ SV Gel kit and PCR Clean-Up System (Promega, Madison, United States) and sent to the DNA Synthesis and Sequencing Facility of the Institute of Biotechnology of UNAM in Mexico for sequencing. The forward and reverse 16S rRNA sequences were assembled and deposited in the GenBank^1^ database under the accession numbers MT002753 (X29) and MT002754 (F51).

Sequences of endophytic actinobacteria, including the reported *S. arenicola* strains (phylotype A and type strain ATCC_BAA-917), were obtained from the NCBI database^2^. Representative microorganisms were selected based on a nearest reported sequence (neighbor) determined by a BLAST (Basic Local Alignment Search Tool; Altschul et al., 1990) analysis with the MT002753 and MT002754 sequences. Multiple sequence alignments were generated using Clustal X (Larkin et al., 2007). The phylogenetic relationships were analyzed using the neighbor-joining method, as implemented in MEGA7 with evolutionary distances computed using the p-distance method (Nei and Kumar, 2000; Kumar et al., 2016). We used 1000 bootstrap replicates for tree support and *Blastococcus litoris* and *Propionibacterium damnosum* as outgroups.

*Azospirillum brasilense* (ATCC 29710), a known terrestrial bacteria with plant growth promotion activity, was preserved on TYG-agar medium (Bashan et al., 2011). For seed-treatment cultures, *A. brasilense* was inoculated into 50 mL of TYG-broth medium and grown at 210 rpm and 28°C for 12 h prior to being introduced into *N. attenuata* seeds.

All plant experiments were conducted with a 31st inbred generation of *Nicotiana attenuata* Torr. ex S. Wats. (a.k.a. wild tobacco; seeds collected from DI Ranch, Motoqua, United States; Baldwin, 1998).

#### Germination Assays

*Nicotiana attenuata* germination is known to follow burn-soil and gibberellic acid (GA_3_) signals (Baldwin and Morse, 1994), which allowed us to coordinate the onset of germination using smoke-cues and GA_3_ to arrest seed dormancy. *N. attenuata* seeds were sterilized for 5–7 min in a 1 mL hypochlorite (2%) aqueous solution supplemented with 10 μL of 0.5% (v/v) Tween-20 (Sigma-Aldrich, St. Louis, MO, United States). Seeds were washed three times with sterile distilled water and incubated for 1 h in 1 mL of sterile 50-fold diluted liquid smoke (House of Herbs, Inc., United States) supplemented with 100 μL of 0.01 M GA_3_ (Biogib, Arysta LifeScience, Mexico). Then, seeds were triple rinsed in sterile distilled water and treated for 30 min with 1 mL of the bacterial suspensions of *S. arenicola* X29, F51, *A. brasilense* (cultured as described in the “Biological Samples” section), or A1 growth media (control). After which, the seeds were sown in plates with solid plant growth media with or without 100 mM NaCl (Sigma-Aldrich). Solid plant growth media contained 0.6% (w/v) phytagel (Sigma-Aldrich, St. Louis, MO, United States) supplemented with Gamborg’s B-5 Basal Medium with Minimal Organics (Sigma-Aldrich). The plates were maintained in a growth chamber (Thermo Scientific) at 28°C with a 16:8 h light/dark photoperiod (155–300 μm/s/m^2^). Germination was evaluated for the next 18 days after sowing.

The experimental units were arranged in a two-level factorial fixed design with the first and second factors being the salinity and bacteria treatments, respectively. A total of eight treatments were used, from which two served as the negative controls. A total of 100 seeds distributed on four plates were tested for each treatment. Contaminated seeds were not included in the sample size. Statistical differences among treatments and conditions were determined by a two-way permutational ANOVA (α = 0.05; 10,000 residual permutations) under a reduced model based on a Euclidean distance matrix in the PRIMER + PERMANOVA software (v.7; PRIMER-e, Plymouth, United Kingdom).

### Plant Growth Analysis

The growth-promoting effect was evaluated by treating the seeds as described (see section “Germination Assay”) with *S. arenicola strains* (X29 and F51), *A. brasilense*, or A1-growth media (negative control). Eight seeds were sown on one side of a square cell culture plate containing solid plant growth media. A total of 40 seeds were used for each treatment. The plates were placed on a vertical plane with the seeds on top. Pictures were taken of the cultured seedlings every day after germination with a scale in frame, and growth was measured using the image processing and analysis software ImageJ v. 1.51 (National Institutes of Health). Significant differences were analyzed by two-way ANOVA (α = 0.05) followed by a Tukey HSD test between the treatments and control.

### Photosynthetic Performance of *Nicotiana attenuata* Plants

The photosynthetic performance of the plant *N. attenuata* was evaluated by non-intrusive pulse-amplitude modulated chlorophyll fluorometry (Junior-PAM, Heinz Walz GmbH, Effeltrich, Germany) according to Schreiber (2004). To gain an overview of photosynthetic performance, we conducted rapid light-curve experiments that allowed us to determine the maximum electron transfer rate (ETR_MAX_) as a proxy of the photosynthetic rate, the maximum photochemical quantum efficiency of photosystem II (PSII; *F*_V_*/F*_M_) as a measure of the health of the photosynthetic apparatus, and the non-photochemical quenching of PSII Chlorophyll *a* fluorescence (NPQ) as a proxy to estimate photoprotective capacity. Experiments were conducted on the same set of *N. attenuata* plants (22 DAG) used in the plant growth analysis, with at least eight plants per treatment analyzed. Plants were submitted to two light regimens: artificial low light (PAR 300 μmol photon m^–2^ s^–1^) and natural high light conditions (∼2,000 μmol photon m^–2^ s^–1^). Plants were exposed for at least 3 h to the respective light conditions and dark acclimated for 30 min prior to the analysis. Statistical differences were determined by two-way ANOVA and pair-wise differences were determined by a Tukey HSD test.

### Microscopic Analysis of Roots

Roots of *N. attenuata* were harvested 22 days after sowing. Roots were heat-fixed, clarified in 70% (v/v) EtOH-KOH for 24 h, and gram stained to visualize root-associated bacteria. Stained bacteria were then observed under a Primo Star compound light microscope (Carl Zeiss, Göttingen, Germany).

### Isolation of *Salinispora* From *N. attenuata* Roots

At the end of the experiments, *N. attenuata* roots were harvested under sterile conditions, homogenized softly with liquid media using a sterile mortar and pestle, and plated on A1 medium to confirm the presence of *Salinispora* strains in the *N. attenuata* roots. *Salinispora* strains were identified by their morphological features.

### Statistical Analysis

Normality and homogeneity of variance were evaluated for all data sets. If the assumptions were fulfilled, the data was analyzed by a one or two-way ANOVA and Tukey HSD tests to compare means of the different treatments and conditions. If assumptions were not fulfilled, permutational ANOVAs were used. Statistical analysis was conducted in Sigma Plot v. 12 (Systat Software, Inc.), R studio (Version 1.2.5033, with R version 3.16, © 2009–2019 RStudio, Inc.) and PRIMER + PERMANOVA software (Version 7.0.13; PRIMER-e, Plymouth, United Kingdom).

## Results

### Isolation and Identification of *Salinispora* spp. as Coral-Associated Bacteria

**FIGURE 2:**
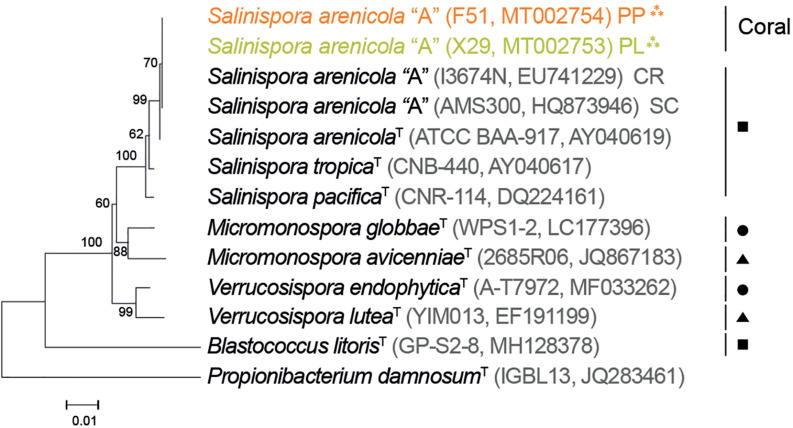
Phylogeny relationship between the coral-isolated strains, *Salinispora*, *Micromonospora*, and *Verrucosispora.* The phylogenetic tree generated from 16S rRNA gene sequences for the neighbor-joining analysis with 1,000 bootstraps. *Blastococcus litoris* and *Propionibacterium damnosum* were used as outgroups. The tree is drawn to scale, with branch lengths measured in the number of base differences per site (strain, accession numbers in parentheses). T, type strain. ***, strains observed in this study. Isolation source: PP, *Porites panamensis*; PL, *Porites lobate*; SC, Sea of Cortez; CR, Costa Rica, ▲ = root mangrove, ■ = marine sediment, • = root land.

Corals form associations with several microorganisms, including Actinobacteria species, known to reside outside and around the mucus layer as well as inside of coral tissues (e.g., on the gastrodermal layer of polyps). By carefully removing the mucus layer and harvesting the clean tissue (see the “Marine Biological Samples and Microorganism Culture” subsection in the “Materials and Methods” section) of *P. lobata* and *P. panamensis* corals, we were able to isolate actinobacteria from coral gastrodermal tissues. These actinobacteria were grown in plates with and without seawater, isolated, and characterized morphologically. Among the actinobacteria that grew strictly in seawater, we were able to identify two putative *Salinispora* strains, X29 from *P. lobata* and F51 from *P. panamensis.* The identity of X29 and F51 was further confirmed by 16S rRNA sequencing. The NJ-phylogenetic tree shows the 16S rRNA sequence similarity of the actinobacterias strains isolated in this work with the previously reported *S. arenicola* phylotype A (100%) and the type strain ATCC_BAA-917 (99.93%) (Jensen and Mafnas, 2006; Becerril-Espinosa et al., 2013; Millan-Aguinaga et al., 2017) (Figure 2). Within this analysis, we also observed a close relationship with other plant-endophytic bacteria from other terrestrial and marine genera (Figure 2).

*Salinispora arenicola* is considered a strict marine bacterium, whose ecological role and relevance is still largely unknown. However, *S. arenicola* has recently gathered considerable attention due to its high proportion of specialized metabolite biosynthetic genes with promising biotechnological potential (Amos et al., 2017; Bauermeister et al., 2018).

### Inoculation of *N. attenuata* Seeds With *S. arenicola* Strains Enhanced Plant Germination Under Saline Conditions

**FIGURE 3:**
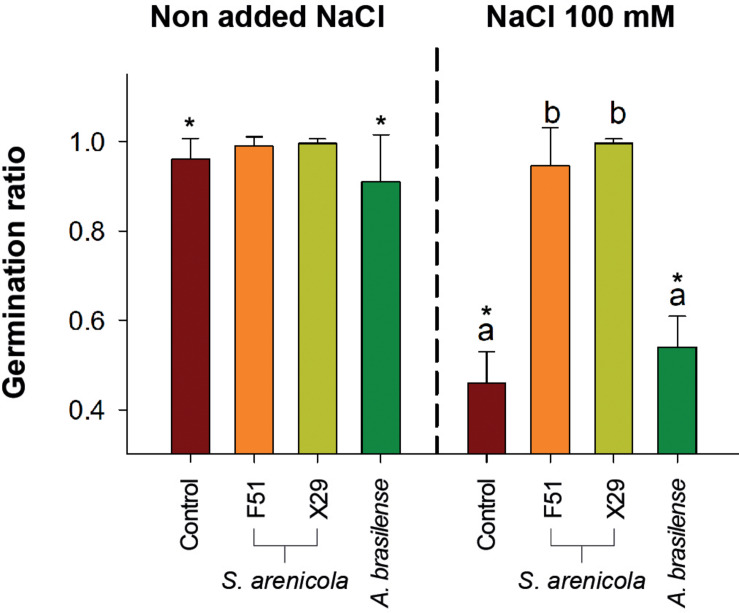
Germination ratio of *N. attenuata* treated with the coral bacteria *S. arenicola* under saline stress. Seeds were treated with either *S. arenicola* strain X29 (yellow), F51 (orange), *Azospirillum brasilense* (green), or growth media (Control; red) and sown in plates with or without with 100 mM NaCl added. Results are the average of at least three independent experiments (±SD). Differences among germination ratios were analyzed by a permutational ANOVA [α = 0.05, *p* < 0.001] followed by pairwise test between the treatments and Control. Lowercase letters to denote statistical differences among treatments in each condition. The symbol (*) denotes statistical differences of the interactions between Treatments × conditions (*p* < 0.001).

Since *Salinispora* is a strict marine bacterium, we hypothesized that it would possess biostimulant properties to cope with salt stress. Therefore, we tested *S. arenicola* activity during the germination of *N. attenuata* seeds *in vitro* under normal and saline conditions (100 mM NaCl). Seeds were treated with either one of the two varieties of *S. arenicola* (i.e., X29 or F51), a known biostimulant bacterium *A. brasilense*, or with sterile liquid growth media as a negative control. Treated seeds were sown in culture plates with and without NaCl (see the “Plant Material, Germination, and Growth Assay” subsection in “Materials and Methods” section), and germination was recorded. Under non-saline conditions, differences in germination were barely observed between the *S. arenicola* strains and that of the control (Figure 3), with only a slight but not significant increase observed in the germination of seeds treated with X29 between days 7 and 10. However, under conditions of increased salinity, we observed significant differences in the germination ratio among seeds treated with either the X29 or F51 strains and those of the control or *A. brasilense-*treated seeds (*P* < 0.001; Figure 2). Under conditions of increased salinity, the germination success at the end of the experiment (day 18) of seeds treated with *S. arenicola* was at least twofold higher with F51 (germination rate, GR: 0.95 ± 0.09) and X29 (GR: 1 ± 0.0) than *A. brasilense* (GR: 0.54 ± 0.07) or the growth-media control (GR: 0.46 ± 0.07). Interestingly, seed treatment with the broadly used biostimulant *A. brasilense* had no effect on germination under any conditions (Figure 3). We further tested the growth-promoting activity of these treatments under the same conditions and found no significant increase in the growth rates among the different treatments (Supplementary Figure S2).

Overall, these results highlight the potential capacity of the X29 and F51 strains of the marine actinobacteria *S. arenicola* to induce resilience in seed germination in saline environments. Even so, the observed responses remain to be tested under field conditions and in different plant systems.

### Treatment of Plants With Marine Bacteria Improves Photosynthetic Responses Under Saline and High Light Conditions

**FIGURE 4:**
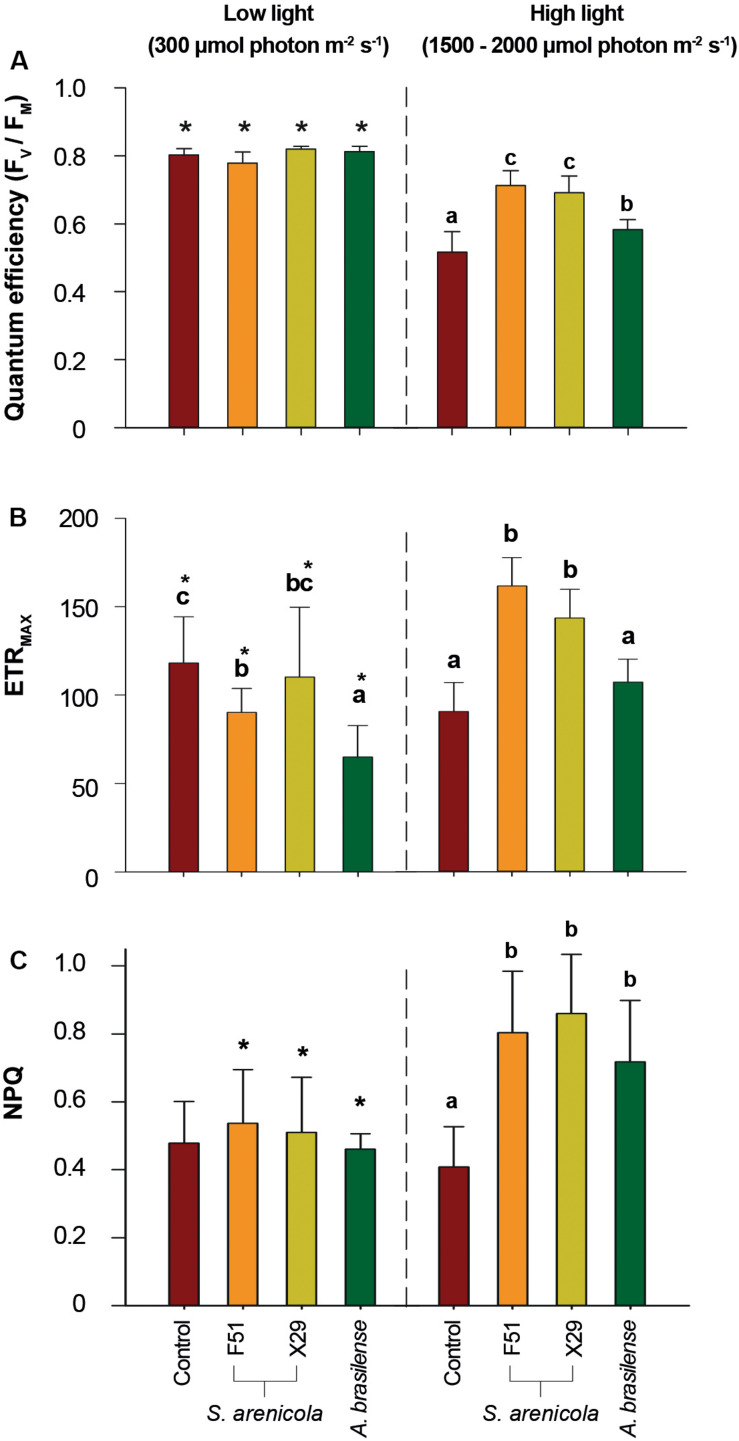
Photosynthetic performance of *N. attenuata* plants grown under saline stress and different light conditions treated with *S. arenicola* bacteria. Plant seeds were treated with bacterial suspensions (*S. arenicola* strain F51, orange; *S. arenicola* strain X29, yellow; *A. brasilense*, green; and growth media Control, red), and the photosynthetic parameters were measured for two light conditions (artificial low light and natural high light). **(A)** *F*_V_/*F*_M_, PSII maximum quantum yield of photosystem II; **(B)** ETR_MAX_, maximum electron transport rate; **(C)** NPQ, non-photochemical quenching of Chlorophyll *a* fluorescence. Results represent the average of eight independent plant measurements. Error bars are standard deviations. Differences were analyzed using a two-way ANOVA (α = 0.05, *p* < 0.001). * indicates differences between conditions among treatments (α = 0.05, *p* < 0.001). Inbox letters highlight differences between treatments for each condition analyzed by Tukey HSD test.

We further explored if the induced resilience to salinity by *S. arenicola* might be observed in other plant physiological responses besides germination. Photosynthetic fluorescence has been suggested to be a hallmark of stress responses (Guidi et al., 2019). We first evaluated photosynthetic responses under culture conditions with relatively low light levels (c.a. 300 μmol m^–2^ s^–1^). We observed no significant differences in the photosynthetic descriptors of *F*_V_/*F*_M_ and ETR_MAX_ in the plants among all treatments after 30 min of dark acclimation when analyzed under low light conditions (Figures 4A,B), suggesting a non-positive effect of these photosynthetic responses.

As it has been observed before (Külheim et al., 2002), it is likely that differences in photosynthetic performance are not always evident under low light conditions. Hence, we tested photosynthetic performance under harsher light conditions. We compared both the photosynthetic performance of *S. arenicola*-treated and untreated plants after exposing them to natural sunlight (1,500–2,000 μmol m^–2^ s^–1^) for c.a. 3 h to induce moderate light stress. Under these relatively high light conditions (Figure 4), we observed significant differences in all photosynthetic parameters among the treatments. We found a clear indication of photoprotection in the *S. arenicola-*treated plants. The PSII quantum efficiency of the plants treated with the *S. arenicola* strains (*F*_V_/*F*_M_ 0.7 ± 0.05) outperformed that of the control plants by 25% (*F*_V_/*F*_M_ c. 0.5 ± 0.06) and that of the *A. brasilense*-treated plants by 15% (*F*_V_/*F*_M_ c. 0.6 ± 0.05; Figure 4A).

Changes in ETR_MAX_ followed a similar pattern as those of the PSII quantum efficiency. *Salinispora* strain F51- and X29-treated plants presented 88 and 68% higher ETR_MAX_ values than those of the control plants, respectively, which was also surprisingly true for *A. brasilense* (48 and 66%, respectively; Figure 4B). These findings suggest that the photosynthetic rate was greater in the marine bacteria-treated plants than in the plants of the other treatments. Higher photosynthetic rates are often correlated to increased growth rates. However, no differences were observed in plant growth *in vitro* (Supplementary Figure S2), which might be due to the smooth low light culture conditions, as suggested by the lack of differential photosynthetic responses in Figure 4. The observed changes under high light conditions suggest that differences in growth may be observed under conditions of natural sunlight and salinity; however, this remains to be tested.

The thermal dissipation of energy measured as NPQ is the principal photoprotective mechanism in plants used to avoid the deleterious effects of excessive light. Interestingly, under low light conditions, no significant differences in NPQ values for the F51, X29, and *A. brasilense*-treated plants were observed. However, under high light conditions, we found that the NPQ values for all *Salinispora*-treated plants were twice that of the control plants, implying a primed capacity of *S. arenicola*-treated plants to overcome excessive light conditions (Figure 4C). Interestingly, NPQ values between *Salinispora*- and *A. brasilense*-treated plants were not significantly different (Figure 4C), which further suggests that the associations between symbiont bacteria and plant roots may prime stress responses that allow the plant to better cope with high light.

Together, these results suggest a photoprotective effect of the marine actinobacteria *S. arenicola* in plants under natural light and saline conditions. This effect was even higher than in the well-known biostimulant and terrestrial bacterium *A. brasilense* under the experimental conditions.

#### *Salinispora arenicola* Formed an Endosymbiotic-Like Relationship With Plant Roots

**FIGURE 5:**
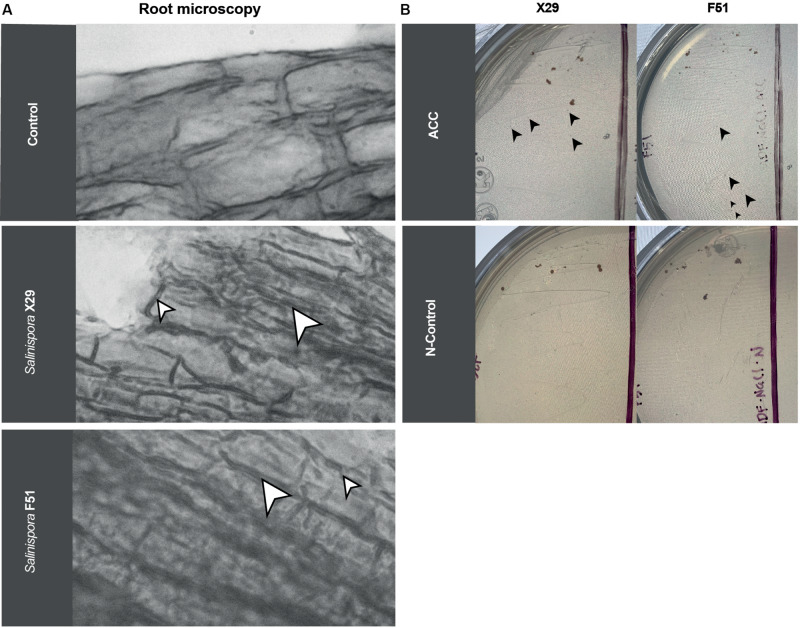
Root endophytic-like interactions and ACC deaminase activity of *S. arenicola* strains on *N. attenuata*. **(A)** Microscopic photography of *N. attenuata* roots grown after seed treatment with *Salinispora* (X29 and F51) strains or sterile growth media (Control). White arrows point to bacteria. To improve the contrast, the images were converted to black and white. **(B)** *S. arenicola* cultured under growth media with nitrogen as the control (N-control) and ACC as the only nitrogen source (ACC). Black arrows point to new colonies.

Biostimulant bacteria are known to interact in different ways with plant roots. Microorganisms grow outside and around the plant epithelium (exophytic) or inside the tissues (endophytic), signaling and providing nutrients. In order to observe the interactions between *N. attenuata* roots and *S. arenicola*, we collected the treated roots, gram-stained them, and analyzed the roots under a microscope. We found that the *S. arenicola* hyphae were highly interwound with *N. attenuata* root apical meristems (Figure 5A), with no bacteria present outside the roots. In contrast, *A. brasilense*, which is known to act as an exophytic symbiont, was mainly observed outside, surrounding the root epithelium (Supplementary Figure S3). The *N. attenuata* seeds treated with media, which served as a negative control, showed no bacteria addition at all (Figure 5A).

These observations suggest an endophytic-like interaction between *N. attenuata* roots and the actinobacteria *S. arenicola*. Moreover, to confirm this observation, we harvested and cultured small cuts of the *N. attenuata* roots on bacteria media under sterile conditions. We were able to re-isolate the *Salinispora* strains (Supplementary Figure S4), which further implies an endophytic growth of the marine bacteria on *N. attenuata* roots.

### *Salinispora arenicola* A Possesses ACC Deaminase Activity

Among the different reported methods that bacteria use to enhance salinity resistance, one of the most studied and recognized is that of ACC deaminase activity by symbiont bacteria. We tested for ACC deaminase activity using the *S. arenicola* strains. Bacteria were inoculated in a medium with only ACC as a nitrogen source and allowed to grow. *S. arenicola* strains were able to grow in nitrogen-restricted media (only ACC), following similar kinetics to those of the nitrogen-rich media, suggesting that *S. arenicola* possesses ACC deaminase activity (Figure 5B). Nonetheless, growth in either media was found to be very slow. From this result, we inferred that at least one mechanism for the observed induced resistance to salinity by *S. arenicola* was present, which might have been either a decrease in stress-related signaling induced by ethylene production (its biosynthesis is prevented by the deamination of ACC) or by the provision of an alternative nitrogen source to the plant.

## Discussion

**FIGURE 6:**
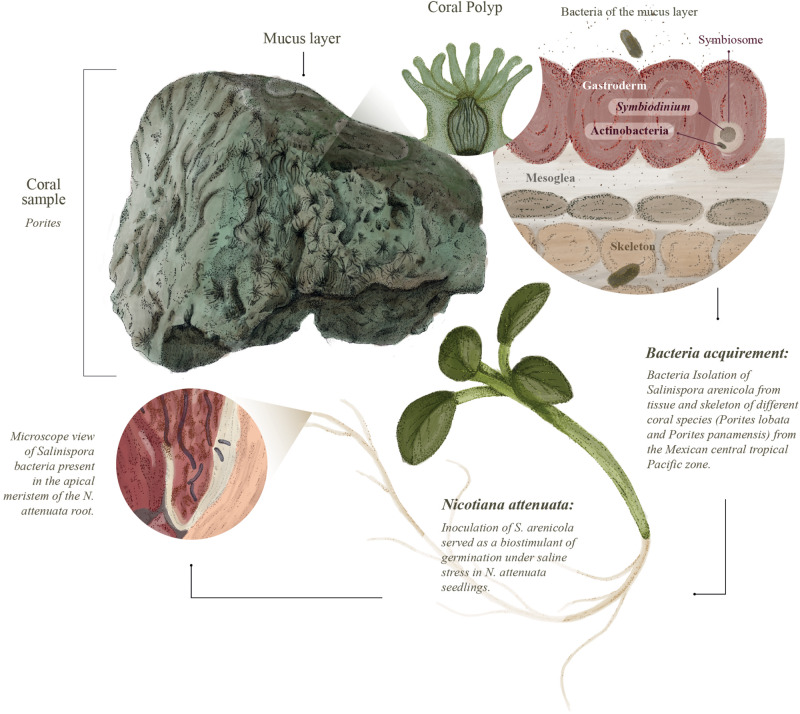
Discovery of coral microbiota with biostimulant activity of land plants. *Salinispora* actinobacteria were isolated from coral polyp tissues of *Porites* spp. harvested from the Mexican tropical central Pacific coast. These Actinobacteria may form associations with other symbionts, such as *Symbiodinium*. We tested the biostimulant activity of the isolated *Salinispora* strains and found a protective effect on *Nicotiana attenuata* plants germinated and grown in saline environments. We also found that the isolated *Salinispora* actinobacteria form endophytic-like interactions with *N. attenuata* roots and likely ACC activity, which may contribute to the observed biostimulant effect.

Here, we address the biostimulant activity of the coral symbiont actinobacteria *S. arenicola* in the solanaceous plant *N. attenuata* (Figure 6). For the first time, we found and isolated *S. arenicola* phylotype A from the associated microbiota of the coral tissues of *P. lobata* and *P. panamensis* (Figures 1, 6). We also observed that *S. arenicola* alleviated salt stress during germination and photosynthesis in *N. attenuata* plants. Our results revealed a positive effect of the actinobacteria in the germination of seeds under salt stress (Figure 3), which was likely through the activity of the ACC deaminase found in *S. arenicola* (Figure 5B). Interestingly, no changes in growth were found among the treatments, possibly because of the low light conditions of the culture (Supplementary Figure S2), as suggested by the photosynthetic responses (Figure 4). Photosynthetic responses were found only to change under high light (sunlight) conditions and not under culture light conditions. Under high light conditions, we observed a photoprotective effect as evidenced by an increase in PSII quantum efficiency (NPQ and ETR_MAX_) in plants treated with *S. arenicola*. Overall, we have shown the potential use of marine resources for the development of alternative sustainable agricultural tools to overcome current and upcoming environmental challenges.

*Salinispora* are strict saline actinobacteria with promising biotechnological potential, which is supported by their high proportion of biosynthetic genes (c.a. 10% of their genome) for specialized metabolites, some with unique chemical structures (Mincer et al., 2002; Udwary et al., 2007; Penn et al., 2009; Prieto-Davó et al., 2016; Amos et al., 2017). Like many other coral-associated bacteria, the specific ecological functions of *Salinispora* in the coral are unknown. It has been proposed that actinobacteria may influence bacterial community structure and protect corals from pathogens by releasing antibiotics (Mahmoud and Kalendar, 2016). Recently, it was shown that *Salinispora* produces Staurosporine, a potent antibiotic, in their native sediments, actively influencing the microbial community assemblage (Patin et al., 2017; Tuttle et al., 2019). Therefore, in the corals *P. panamensis* and *P. lobata*, the isolated *Salinispora* strains may exert a defense-like function against pathogens. However, due to the great extent of specialized secondary metabolites with unknown functions, *Salinispora* probably exerts some other ecological functions in corals.

There is some evidence that habitat-adapted symbiotic bacteria isolated from halophytes enter into symbiosis with many other terrestrial plants, including crops, and confer the capacity to grow under salt stress conditions (Rodriguez et al., 2008; Redman et al., 2011; Albdaiwi et al., 2019). The mechanisms associated with salt stress reduction in plants induced by these halophilic bacteria are varied (i.e., production of ACC deaminase, activation of antioxidant enzymes to eliminate reactive oxygen species, improvement of plant nutrition, production of phytohormones, accumulation of osmolytes, changes in the root architecture and hydraulic conductance, and the increased synthesis of chlorophyll and pigments to preserve photosynthetic activity; Etesami and Beattie, 2018). Recently, it has been reported that actinobacteria isolated from the marine environment enhanced germination in plants grown under high salinity conditions. This result was further linked to the activity of ACC deaminase (Qin et al., 2009, 2014; Gong et al., 2018). It is believed that ACC deaminase redirects ACC metabolism away from ethylene production, avoiding ethylene root growth inhibition and instead providing nitrogen (Van de Poel and Van Der Straeten, 2014). Our results (Figure 5B) suggest that ACC deaminase-like enzymes could be present in the symbiotic *Salinispora* strains assayed since we found the growth of *Salinispora* in culture medium with ACC as the only source of nitrogen. Therefore, ACC deaminase could be one of the factors that exerts a positive influence on germination and photosynthesis in plants under salt stress conditions. However, there are no reports of *Salinispora* owning an ACC deaminase gene nor did we find a reported putative homolog among the different genome databases (Integrated Microbial Genomes and Microbiomes^3^; NCBI^4^). We did find a PALP domain-containing protein^5^ with similarities to D-cysteine desulfhydrase and ACC deaminase from related actinobacterial taxa (*Nonomuraea wenchangensis* and *Streptomyces thermoautotroph*, 54.5 and 48.1% similarity, respectively^6^). Future research is needed to reveal the specific ACC deaminase-like enzyme or the other fundamental molecular mechanisms behind the observed responses.

In contrast to terrestrial actinobacteria, *Salinispora* acquired genes during evolution that conferred the ability to adapt to the marine environment, highlighting light electron transport, sodium and ABC transporters, and channels and pores (Penn et al., 2009; Penn and Jensen, 2012). Possibly, mechanisms for stress alleviation exerted by *Salinispora* may be related to the more than 50 acquired genes for marine adaptation, like genes for specific ion transporters that contribute to decreasing salinity stress and the release of many osmolytes.

We also found that the interaction between *N. attenuata* and *S. arenicola* resembled that of plant-endophytic symbiosis (Figure 5A). Other actinobacteria (i.e., *Micromonospora* and *Verrucosispora*) from the same family of the coral symbiont *Salinispora* have been isolated from the root nodules of terrestrial and marine plants where they presented endophytic interactions and conferred advantages to their plant hosts (Figure 2; Li et al., 2013; Ngaemthao et al., 2017; Kuncharoen et al., 2018). Bioinformatic analysis suggests that *Salinispora* spp. have evolved from this terrestrial *Micromonospora*, with many of the metabolic genes being conserved (Trujillo et al., 2014). Likely, *Salinispora* conserves the capacity to establish plant-endophytic relations and some of their plant-biostimulant properties. Interestingly, the plant-biostimulant activity observed by *S. arenicola* was different from that reported for *A. brasilense.* The bioactivity of *A. brasilense* is based on the production of the phytohormone IAA. Screening of *S. arenicola* extracts revealed no traces of IAA (Supplementary Figure S5). Moreover, *A. brasilense* did not affect germination and only exerted a mild effect on photosynthetic performance under saline stress (Figure 4).

Improvement in photosynthetic performance in plants under saline stress has been associated with the overproduction of chlorophyll and carotenoid pigments as well as an increase in antioxidant enzymes. For example, in *Maiz* seeds, biopriming with the halotolerant *Pseudomonas geniculata* was found to increase chlorophyll and carotenoid pigment content (c.a. 50%) as well as antioxidant enzyme activity (c.a. 150%; Singh et al., 2020). Recently, IAA has been shown to specifically up-regulate chlorophyll content via Auxin response factor 6A (Yuan et al., 2019), which may account for the mild positive effect on the photosynthetic performance of *N. attenuata* plants treated with *A. brasilense* under conditions of saline stress. However, the photosynthetic performance induced by *A. brasilense* was easily surpassed by the effect of *S. arenicola* in plants (Figure 4).

Similarly, increased pigment content may be expected in *Nicotiana* plants when treated with *Salinispora*, although through a different mechanism. Saline stress activates ethylene biosynthesis and signaling, which is also known to reduce photosynthesis in young leaves while promoting plant senescence (Tholen et al., 2008; Ceusters and Van de Poel, 2018). The discussed ACC deaminase activity by *S. arenicola* may also explain the enhanced photosynthetic responses under saline stress, by either decreasing ethylene stress-related photosynthetic signaling or by providing an alternative nitrogen source and thus boosting photosynthetic performance. Experiments with the direct supplementation of ethylene and impaired ethylene signaling in plants are required to resolve the mechanism behind this response.

Despite the c.a. 400 million years of independent evolution and contrasting habitats between marine and terrestrial ecosystems, research has shown essential similarities in the chemical cues that regulate ecological interactions (Rasher et al., 2015). This is further supported by the surprisingly well conserved metabolic-related ortholog genes observed in *S. arenicola* and their terrestrial counterparts (Trujillo et al., 2014). In this study, we observed the capacity of this marine actinobacteria to establish an endosymbiotic-like interaction with terrestrial plants (Figure 5). However, there are important differences among the organisms of these two ecosystems, which were highlighted by the addition of a vast number of metabolic genes in *S. arenicola* that are different from those of their terrestrial counterparts (Penn et al., 2009; Penn and Jensen, 2012; Trujillo et al., 2014). These differences may provide alternative biotechnological solutions, such as the one reported here.

Plant- and seed-borne endophytes provide beneficial attributes to plants by reciprocally allowing a closer exchange of resources and signals between organisms (Truyens et al., 2015). While plants are responsible for primary production in the terrestrial sphere, the photosynthetic endosymbionts of corals are the key producers in reef communities. Both plants and algae respond to similar chemical and biochemical cues of a related nature, such as phytohormone IAA and possibly ACC deaminase. As much more is known about the chemical ecology of terrestrial ecosystems, here we posit the need to better understand marine ecological interactions as an important alternative source for future discoveries to overcome current environmental and agricultural challenges.

The present report shows the use of *S. arenicola* as a terrestrial plant biostimulant with the potential to alleviate germination and photosynthesis under saline stress, which is a great potential benefit for crop plants. However, further research on the capacity of *S. arenicola* to enhance crop yield and plant growth should be conducted to uncover the full potential benefits of this coral bacteria. Furthermore, although endosymbiosis and the biostimulant activity of plant growth promoting bacteria are known to occur in many related species, our research was focused on one terrestrial plant, which may or may not be reproducible in other crop-plant systems. Future experiments should evaluate this activity in related crop plant species, such as in tomato plants. Moreover, studies of the innocuity of the bacteria and ecological safety are required as well as of the efficiency of formulations. In addition, an economic cost-benefit analysis will be required. Therefore, the pursuit to discover the potential uses of marine organisms for land-agriculture solutions is both promising and extensive.

## Conclusion

Soil salinity is one of the most damaging environmental stressors worldwide that is responsible for significant reductions in croplands, crop productivity, and crop quality. Our results provide evidence of the use of the marine actinobacteria *S. arenicola* as an alternative to counteract the adverse effects of soil salinity on the germination and photosynthetic performance in plants. Although environmental issues and field conditions remain to be analyzed, the reported plant biostimulant activity of *S. arenicola* can easily be scaled up for crop use, as it has been done for other organisms, such as *A. brasilense*.

Historically, actinobacteria have distinguished themselves as organisms with great biotechnological potential for the production of antibiotics and other compounds. However, there is still much to explore in this phylum and in other marine microorganisms. We do not rule out whatsoever that the properties of *S. arenicola* that are described here are only a small part of their biotechnological potential, such as the production of novel specialized metabolites. Certainly, a better understanding of the ecological interactions of marine microorganisms, such as those in coral reefs, will provide novel tools to meet current and future environmental and agricultural challenges. A hidden marine treasure awaits discovery.

## Data Availability Statement

*Salinispora arenicola* strains X29 and F51 sequences can be found in the NCBI database under the accession numbers MT002753 and MT002754, respectively.

## Author Contributions

HO-A, IM-C, and AB-E conceived and designed the experiments. HO-A, IM-C, CM-S, AM-U, FC-L, and AB-E performed the experiments and interpreted the results. HO-A, IM-C, FR-Z, and AB-E analyzed the data. CM-S designed and elaborate the figures with contributions of HO-A, IM-C, RH-H, and AB-E. HO-A, IM-C, and AB-E wrote the text with contributions of ER-J, FR-Z, CR-M, AM-U, RH-H, and FC-L. All authors approved the final version of the manuscript.

## Conflict of Interest

The authors declare that the research was conducted in the absence of any commercial or financial relationships that could be construed as a potential conflict of interest.

## Footnotes

1 http://www.ncbi.nlm.nih.gov/genbank/index.html

2 http://www.ncbi.nlm.nih.gov/

3 https://img.jgi.doe.gov/

4 https://www.ncbi.nlm.nih.gov/

5 https://www.uniprot.org/uniprot/A0A2W2CP37

6 https://www.uniprot.org/blast
