# Supplementary material for: Diving Into Reef Ecosystems for Land-Agriculture Solutions: Coral Microbiota Can Alleviate Salt Stress During Germination and Photosynthesis in Terrestrial Plants
Source: Front Plant Sci. 2020 May 25;11:648. doi: 10.3389/fpls.2020.00648 (PMC7261865; doi:10.3389/fpls.2020.00648)
Supplement: Supplementary file 1 [file Presentation_1.pdf]

## *Supplementary Material*

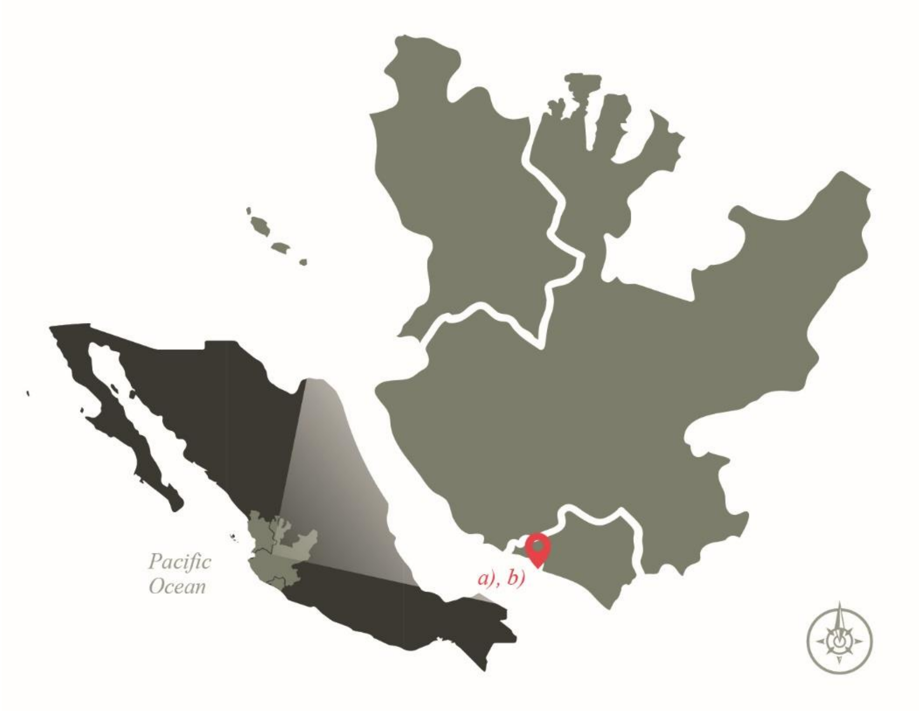


**Supplementary Figure 1**. Collection of coral samples from *Porites lobata* and *Porites panamensis.* Branches of living corals were collected by scuba diving in two coral reefs from the tropical central Pacific: 19° 5' 55.21" N, 104° 23' 24.47" W (*Porites lobata*) and 19° 3' 28.87" N, 104° 15' 40.25" W (*Porites panamensis*).

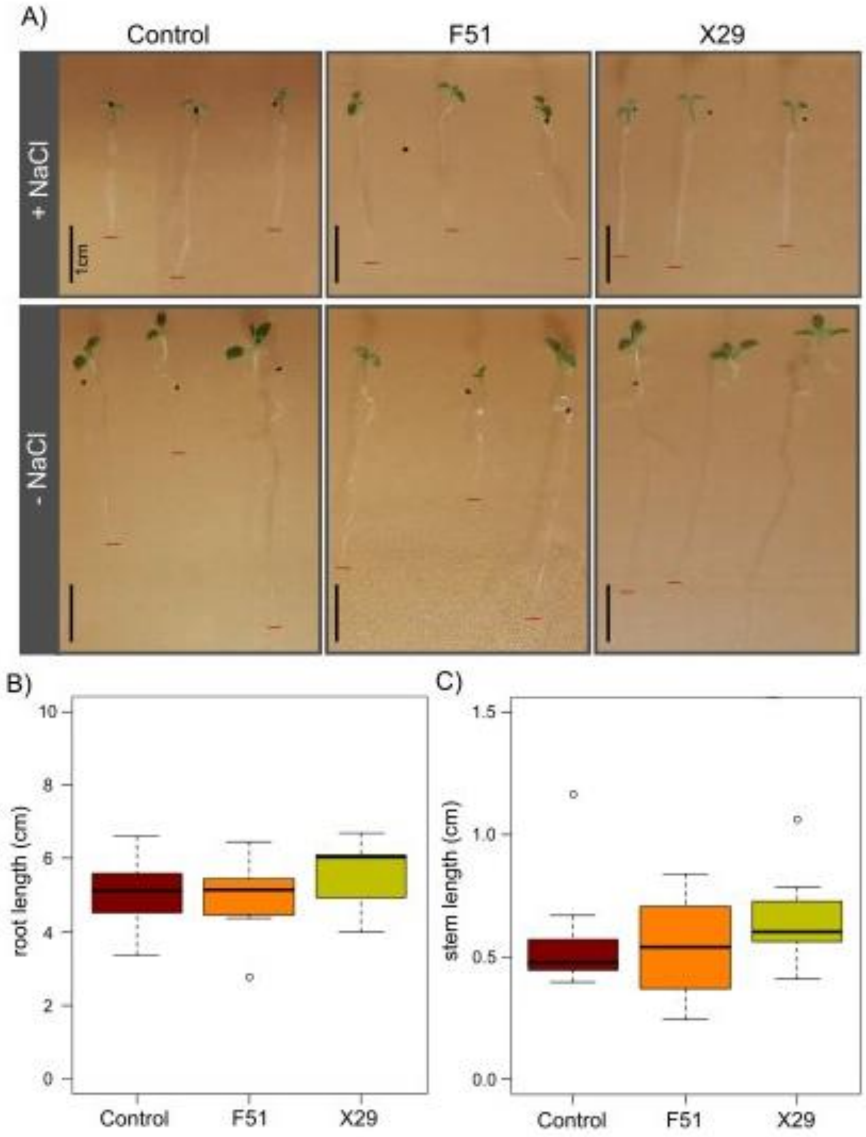


**Supplementary Figure 2.** Bacterial seed treatments did not promote changes in plant growth under saline conditions. (A) Pictures of 15 -grown seedlings after seeds were treated with *Salinispora* strains (F51 or X29) and those of the untreated control grown on media with NaCl (+NaCl) or without (-NaCl). Red lines indicate the end of the primary root. Root (B) and stem (C) length were measured using the image processing and analysis software ImageJ. Data correspond to 18 DAG. No significant differences were observed [ANOVA, α=0.05, (B) p = 0.164, (C) p= 483].

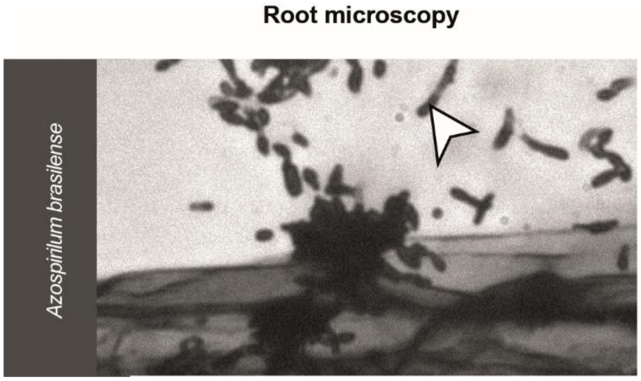


**Supplementary Figure 3.** Exobiotic interaction of *Azospirillum brasilense* with *Nicotiana attenuata* roots. Microscope photograph of *Nicotiana attenuata* roots grown after seeds were treated with *A. brasilense.* White arrows point to bacteria.

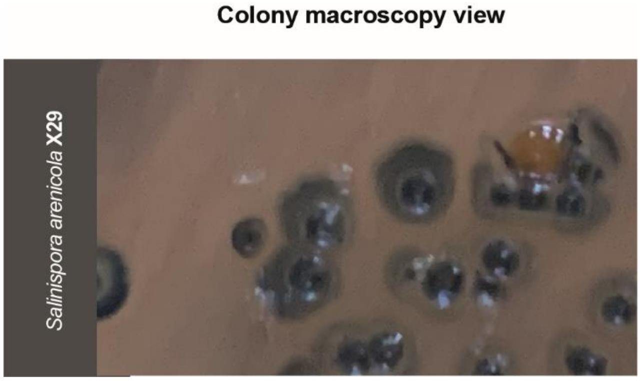


**Supplementary Figure 4**. *Salinispora arenicola* strains isolated from the *Nicotiana attenuata* roots of seeds treated with the X29 strain.

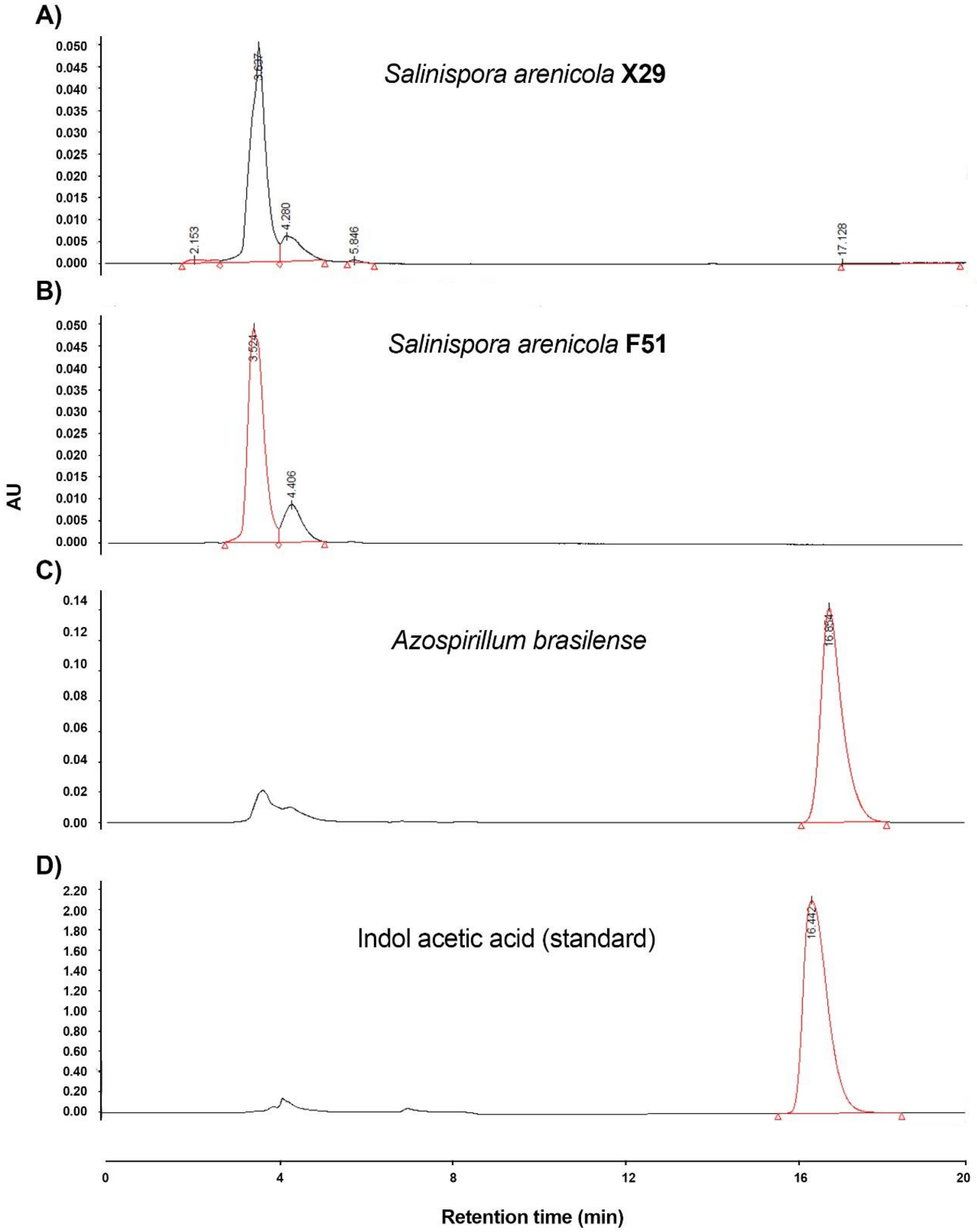


**Supplemental Figure 5.** Coral-isolated *Salinispora arenicola* (F51 and X29) strains do not produce indole acetic acid (IAA). Separation and detection of IAA by HPLC coupled to a UV-Vis detector from the bacteria extracts (*Salinispora arenicola* X29 and F51 strains and *Azospirillum brasilense*). Production of IAA [retention time (RT) peak at 16-18 min] was only observed for the terrestrial biostimulant bacteria *Azospirillum brasilense* but not for the X29 or F51 strains. The RT peaks observed for X29 and F51 correspond to an unidentified compound.
